# Cold Ambient Temperature Promotes *Nosema* spp. Intensity in Honey Bees (*Apis mellifera*)

**DOI:** 10.3390/insects8010020

**Published:** 2017-02-09

**Authors:** Gina Retschnig, Geoffrey R. Williams, Annette Schneeberger, Peter Neumann

**Affiliations:** 1 Institute of Bee Health, Vetsuisse Faculty, University of Bern, Bern 3003, Switzerland; williams@auburn.edu (G.R.W.); annette.schneeberger@hispeed.ch (A.S.); peter.neumann@vetsuisse.unibe.ch (P.N.); 2 Agroscope, Swiss Bee Research Centre, Bern 3003, Switzerland; 3 Department of Entomology & Plant Pathology, Auburn University, Auburn, AL 36849, USA

**Keywords:** *Nosema* spp., parasite intensity, temperature, weather, microsporidian infection, *Apis mellifera*

## Abstract

Interactions between parasites and environmental factors have been implicated in the loss of managed Western honey bee (=HB, *Apis mellifera*) colonies. Although laboratory data suggest that cold temperature may limit the spread of *Nosema ceranae*, an invasive species and now ubiquitous endoparasite of Western HBs, the impact of weather conditions on the distribution of this microsporidian in the field is poorly understood. Here, we conducted a survey for *Nosema* spp. using 18 Swiss apiaries (four colonies per apiary) over a period of up to 18 months. Samples consisting of 60 workers were collected monthly from each colony to estimate *Nosema* spp. intensity, i.e., the number of spores in positive samples using microscopy. Ambient apiary temperature was measured daily to estimate the proportion of days enabling HB flight (>10 °C at midday). The results show that *N*o*sema* spp. intensities were negatively correlated with the proportion of days enabling HB flight, thereby suggesting a significant and unexpected positive impact of cold ambient temperature on intensities, probably via regulation of defecation opportunities for infected hosts.

## 1. Introduction

Parasites have been identified as one threat to the health of managed Western honey bee colonies, *Apis mellifera* [1,2,3], which have faced increased winter losses in recent years in the northern hemisphere [4,5]. Such host–parasite systems can be influenced by numerous biotic and abiotic factors (e.g., genetics, host immunity, temperature and climate, respectively, [6,7,8,9]). Environmental conditions can exert a direct impact on the parasite [10,11] or indirectly influence them by altering host behavior, immunity or physiology [12]. This can include parasite prevalence and intensity as two standard measures to describe parasite presence and distribution. While prevalence refers to the proportion of infected hosts, intensity describes the number of parasites living in an infected host [13].

The Western honey bee can act as the host of multiple parasites [1], ranging from ectoparasites such as *Varroa destructor* [14] and *Tropilaelaps mercedesae* [15] to endoparasites, such as various bee viruses [1,14,16,17] and microsporidia *Nosema* spp. [16,18]. Among environmental factors, temperature is a key element for the life cycle of honey bees in temperate regions [19,20]. During the warm spring and summer months, honey bee colonies reproduce, forage and build up energy stores, whereas they spend the cold months overwintering in the hive with a reduced number of adult workers [19]. Because brood requires a constant temperature of 34.5 °C, and because active heating is costly in terms of energy that requires foraging opportunities, the colony is usually broodless during winter [19]. By influencing the annual life cycle of the honey bee colony, temperature can also have a tremendous effect on its parasites. For example, the ectoparasitic mite *Varroa destructor* requires capped honey bee brood cells for reproduction [14]. The temperature-dependent broodless period in colonies has therefore a strong effect on this parasite’s development. In contrast, the impact of weather conditions on endoparasites may be less obvious. The two microsporidians, *Nosema apis* and *Nosema ceranae,* are both obligatory intracellular gut parasites of *A. mellifera* [16,21,22] that are mainly transmitted via the fecal-oral route [16,23]. After intake of *Nosema* spp. spores per os, endothelial midgut cells are infected by the injection of infective sporoplasm via a polar filament, a highly specialized feature for host infection [16,24]. After rapid reproduction of spores in the midgut of an infected honey bee, the spores are discarded with the feces to potentially infect new hosts. While *Nosema* spp. spores encounter fairly regulated temperatures during their life stages within the host, they are exposed to outside temperature in the course of transmission to new hosts. The two *Nosema* species exhibit a differential sensitivity to temperatures, with *N. ceranae* showing better adaptation to elevated temperatures [10,25,26]. However, *N. ceranae* spores lose viability after 1 week at −18 °C, and are more vulnerable to low temperatures compared to *N. apis* [16]. This may be a potential explanation for the observed tendency of *N. apis* to be more prevalent in temperate, northern countries compared to *N. ceranae*, which is more prevalent in sub-tropical, southern countries [16]. While both *Nosema* species have been detected in Swiss honey bee colonies in the past [27,28,29], *N. ceranae* appears to be much more prevalent; in a recent survey, all 29 colonies were infected with only *N. ceranae* [28].

While seasonal patterns, with a characteristic peak in spring, have repeatedly been described for infection with *N. apis* (e.g., [30,31]), there is no conclusive evidence of whether *N. ceranae* follows a similar seasonal cycle (e.g., [29] vs. [32]). A recent study of *N. ceranae* intensities in tropical and subtropical climates of Taiwan has reported one clear peak in winter and a significant negative correlation of infection intensities with temperature [33]. In contrast, no effects of temperature on *Nosema* intensities were found in feral colonies in temperate climates [34]. For *N. apis*, it has been suggested that higher infections in spring might be the result of low winter temperatures in temperate climates [18]. Since honey bees do generally not fly at temperatures below 9 °C [35] and do not forage below 12 °C [19], workers may remain in the hive for numerous weeks or even months during winter. As part of social immunity, honey bees undertake cleansing flights to defecate exclusively outside of the hives [19], presumably to limit the fecal-oral transmission route of parasites. Thus, honey bees can store feces in their rectum for an extended time period until outside conditions allow them to fly [19]. In this context, ambient temperature and the possibility to defecate not only influence *Nosema* spp. prevalence in the colony, but also *Nosema* intensity in the infected honey bees by accumulation of spores in infected individuals [36,37,38]. However, it is not known how honey bee flight days influence *Nosema* spp. population dynamics.

Therefore, the aim of this study was to investigate the potential influence of ambient temperature on *Nosema* spp. intensity in managed honey bee workers. According to the hypothesis that cold ambient temperature might prevent workers from performing defecation flights, we would expect to find higher *Nosema* spp. intensities during lower temperatures. To investigate this question, we monitored *Nosema* spp. intensities in pooled worker samples from 72 Swiss colonies over two years, and simultaneously measured ambient apiary temperatures. The measured midday temperatures were used to estimate flight day proportions for the colonies of each apiary.

## 2. Materials and Methods

### 2.1. Sampling

The study was conducted throughout Switzerland from April 2011 to October 2012 in cooperation with 18 beekeepers (2010–2011: 15, 2012: 18). Each beekeeper was instructed to randomly choose four colonies from the same apiary, and to collect adult worker samples from these same colonies during the entire study period. In case of a colony loss, the experimental colony was replaced with a new one from the same apiary. The sampling took place once during the last week of every month from April to October, and if possible once or twice during winter (i.e., samples Winter I and Winter II). From each colony, the beekeepers sampled ~100 live adult workers from an outer honey frame to avoid the collection of young bees that may not have been exposed to *Nosema* spp. or that may not have had the opportunity to develop a potential infection yet. The workers were sampled in-hive to enable sampling during all weather conditions, as well as during colder months. The samples were stored in freezers maintained at −20 °C, except when transported on ice to the laboratory.

### 2.2. Nosema spp. Spore Quantification and Species Identification

For each sample, pools of 60 randomly selected workers were analyzed to detect an infection level of 5% with a probability of 95% [39,40]. The whole workers were manually crushed with a porcelain mortar in 15 mL of TN buffer (= 10 mM **T**ris 0.4 M **N**aCl, , pH 7.2) using plastic extraction bags (type Universal 12 × 14 cm, Bioreba, Reinach, Switzerland), and stored at −20 °C in 2 mL Eppendorf tubes. *Nosema* spp. spores were quantified under a light microscope (Laborlux K, Leitz, Wetzlar, Germany) using a bright-lined haemacytometer (Thoma, L.O. Labor Optik, Friedrichsdorf, Germany) at a magnification level of 400× according to Cantwell (1970); they were expressed as mean numbers of *Nosema* spp. spores/bee [41,42]. *Nosema* species was identified in a defined subsample (samples from April (2011 *N* = 60, 2012 (*N* = 50)), June (2011 *N* = 55, 2012 *N* = 70) and October (2011 *N* = 38, 2012 *N* = 57) of both years) using PCR. First, DNA was extracted from the suspension using a DNA extraction kit (NucleoSpin^®^ DNA Tissue, Macherey Nagel, Oensingen, Switzerland) following user manual instructions. For PCR, samples were run in duplicate and specific primers were used for the detection of *N. ceranae* and *N. apis* [43].

### 2.3. Temperature and Honey Bee Flight Days

The temperature at each apiary was recorded daily at midday (12.00) from April 2011 to October 2012 using DS1923 Hygrochron dataloggers (iButton from Embedded Data systems, Lawrenceburg, KY, USA). Based on reports that bees usually do not fly <9 °C [35], and only begin to forage at ~12 °C [19], bees were considered able to fly when the recorded midday temperature was above 10 °C. Using this threshold, the proportion of flight days was calculated for each apiary for four time periods (i.e., one, two, three, and four weeks) prior to sampling. Because no dates were pre-defined for the collection of the winter samples, the time intervals for the calculation of flight days were individually adapted to the sampling month of each winter sample. For each of the defined time periods, colonies were then divided into two groups: (1) 100% flight days (i.e., midday temperatures always above threshold of 10 °C) and (2) less than 100% of flight days (i.e., midday temperatures not always above threshold of 10 °C). These categories were selected because of two reasons; first, to investigate whether relatively small differences in flight day proportions can affect parasite intensity and second, to obtain two balanced groups based on the obtained data distribution of flight day proportions (data were not normally distributed, Kolmogorov-Smirnov tests, *p*s > 0.05). The *Nosema* spp. intensities of the two categories were then compared separately for each time period. Furthermore, analyses were conducted for several incremental flight day opportunities (i.e., ≥90% vs. <90%, ≥75% vs. <75%, and ≥50% vs. <50%). Additionally, potential associations of *Nosema* spp. intensities for all flight day thresholds for the four time intervals, as well as the average monthly midday temperatures, were assessed.

### 2.4. Statistical Analyses

Intensity refers to the number of detected spores in the *Nosema* spp. positive colonies expressed as mean number of *Nosema* spp. spores/bee. Since intensity and flight day proportion data were not normally distributed (Kolmogorov-Smirnov tests, all *p*s > 0.05), non-parametric tests were applied. *Nosema* spp. intensity data were compared between sampling months using a Kruskal-Wallis ANOVA, followed by Bonferroni multiple comparison tests. For flight days, the *Nosema* spp. intensities of the infected colonies were compared among two groups (separately for 100% of flight days vs. <100% of flight days, ≥90% vs. <90%, ≥75% vs. <75 of flight days, and ≥50% vs. <50% of flight days) using the Wilcoxon rank sum test. Additionally, comparisons of the 100% flight day threshold were conducted with apiary as the experimental unit. Relationships between *Nosema* spp. intensities and the proportion of flight days of different time intervals were calculated using the Spearman rank correlation. Similarly, a potential association of *Nosema* spp. intensity and average monthly ambient midday temperature of all samples of the entire study period was assessed using the Spearman rank correlation. *p*-values below 0.05 were considered significant. All statistical analyses were carried out using the program NCSS 10.

## 3. Results

In total, *Nosema* spp. spores were detected in 420 out of 900 (46.7%) pooled worker samples investigated (2011: Winter I: *N* = 10, Winter II: *N* = 9, April: *N* = 35, May: *N* = 27, June: *N* = 20, July: *N* = 33, August: *N* = 23, September: *N* = 15, October: *N* = 13; 2012: Winter I: *N* = 7, Winter II: *N* = 3, April: *N* = 32, May: *N* = 40, June: *N* = 46, July: *N* = 37, August: *N* = 29, September: *N* = 25, October: *N* = 16).

### 3.1. Nosema spp. Intensity and Species Identification

**Figure 1:**
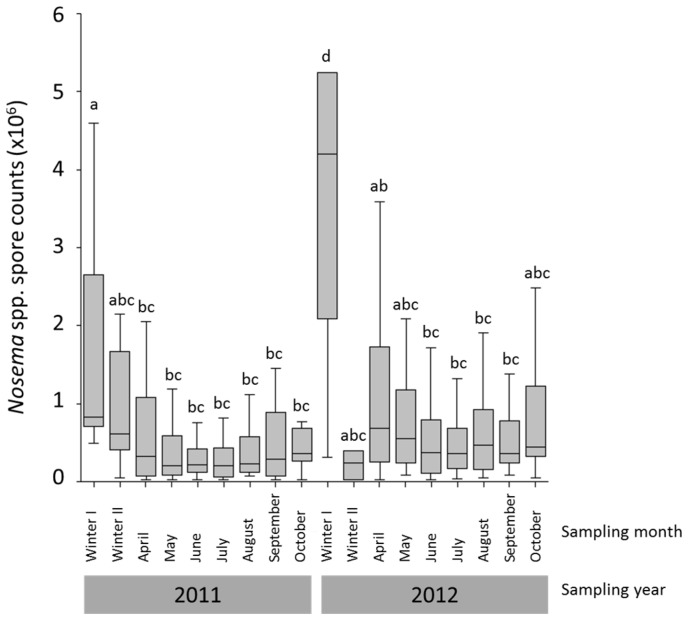
*Nosema* spp. intensities of all infected colonies (*N* = 420) per sampling month (*N* = 3 to 46) over the entire study period. Samples were taken monthly from April to October and once or twice during winter (Winter I and Winter II samples). *Nosema* spp. intensities were compared using Kruskal–Wallis ANOVA and Bonferroni multi comparison tests. Significant differences (*p* < 0.05) are indicated by different letters (a,b,c,d). Boxplots show inter-quartile range (block), Median (black line within box) and data range (vertical lines).

Median *Nosema* spp. intensities of the infected colonies during the whole study period were 0.2–4.2 × 10^6^ spores/bee (Figure 1, Supplementary Material Dataset 1 *Nosema* spp. intensity). On the apiary level, the median spore intensities for the sampling months ranged from 0.17 to 2.75 × 10^6^ spores/bee. The comparison of *Nosema* spp. intensities for the different sampling months showed significantly higher values in the Winter I samples (median: sample Winter 1 2011: 0.825 × 10^6^ spores/bee, Winter 1 2012: 4.2 × 10^6^ spores/bee) than in 11 other sampling months (Winter I sample from 2011) and in all other sampling months (Winter I sample from 2012), respectively (Kruskal-Wallis ANOVA, Bonferroni multiple comparison tests, *p* < 0.05, Figure 1). Additionally, higher *Nosema* spp. intensity was detected in April 2012 compared to July 2011 (Kruskal-Wallis ANOVA, Bonferroni multiple comparison test, *p* < 0.05), but samples from April 2011 and Winter II, May, June, July, August, September and October from both years showed no significant differences (Kruskal-Wallis ANOVA, Bonferroni multiple comparison tests, *p* > 0.05, Figure 1).

In the analyzed subsample, *Nosema* spp. was detected in 57.6% of the samples. Of the *Nosema* positive samples, 89.47% of the samples contained *N. ceranae* only, 4.21% of the samples *N. apis* only and 6.32% both *Nosema* species.

### 3.2. Association of Flight Days with Nosema spp. Intensity

**Figure 2:**
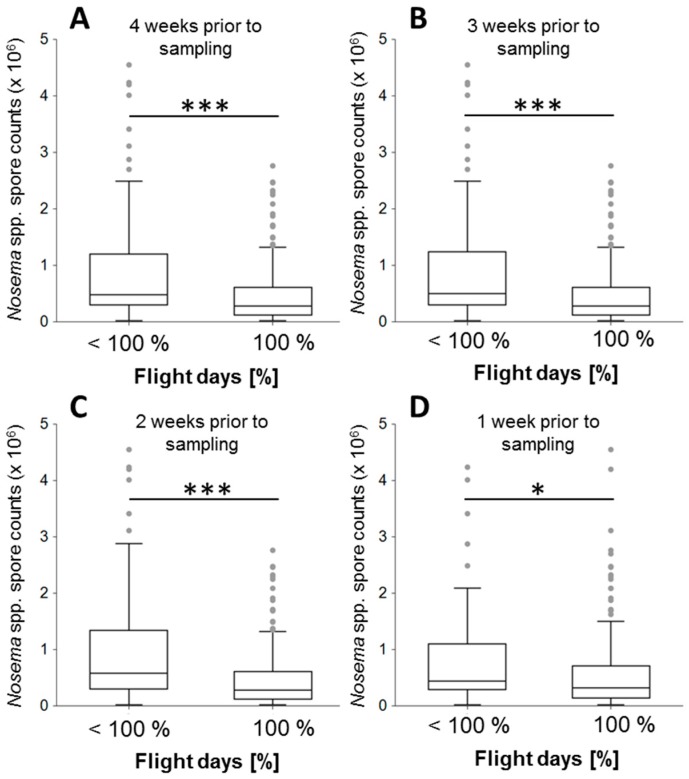
*Nosema* spp. intensities and the proportion of days enabling honey bee flight (>10 °C) in two categories: Colonies that had less than 100% of flight days are compared to colonies that had 100% of flight days and are separately displayed as boxplots for four (**A**); three (**B**); two (**C**) and one (**D**) week prior to the sampling. For each time period, *Nosema* spp. intensities were significantly higher in the group with less than 100% of flight days (Wilcoxon rank sum tests, *** = *p* < 0.001, * = *p* < 0.05). Boxplots show inter-quartile range (block), Median (black line within box), data range (vertical lines) and outliers (grey dots).

Considering the measures that were taken during 4 weeks prior to sample collection, 74.27% of the colonies from which samples were collected experienced 100% flight days, while 25.73% were assigned to the group with less than 100% flight days. In the latter group, the median flight day proportion was 68%, and quartiles Q1 and Q3 were 53% and 92%, respectively. Comparison of *Nosema* spp. intensities in the colonies that experienced 100% flight days to those with less than 100% showed significantly higher *Nosema* spp. intensities in the colonies with less than 100% of flight days for all investigated time periods (Wilcoxon rank sum test, four, three and two weeks prior to sampling: all *p*s < 0.0001, one week prior to sampling: *p* < 0.023, Figure 2). To test whether these results were influenced by the high intensities in the Winter I samples, analyses were repeated with the winter months excluded. This revealed that the *Nosema* spp. intensities between the two groups were still significantly different (four-two weeks: *p* < 0.0001, one week: *p* < 0.015).

Comparisons of *Nosema* spp. intensities in colonies using 90% and 75% flight day thresholds showed significantly higher *Nosema* spp. intensities in the colonies experiencing flight days below these thresholds compared to above for all investigated time periods (Wilcoxon rank sum test, four, three and two weeks prior to sampling: all *p*s < 0.01, one week prior to sampling: *p*s < 0.033, Table 1). No significant difference in *Nosema* spp. intensity occurred at the 50% flight day threshold (Wilcoxon rank sum test, four, three, two and one week prior to sampling: all *p*s > 0.066, Table 1).

With apiary as the experimental unit for the 100% flight day threshold, *Nosema* spp. intensities were significantly lower when flight day proportions were 100% compared to less than 100% at four, three and two weeks prior to sampling (Wilcoxon rank sum test, all *p*s < 0.01), but not at one week prior to sampling (Wilcoxon rank sum test, *p* > 0.2, Table 1).

**Figure 3:**
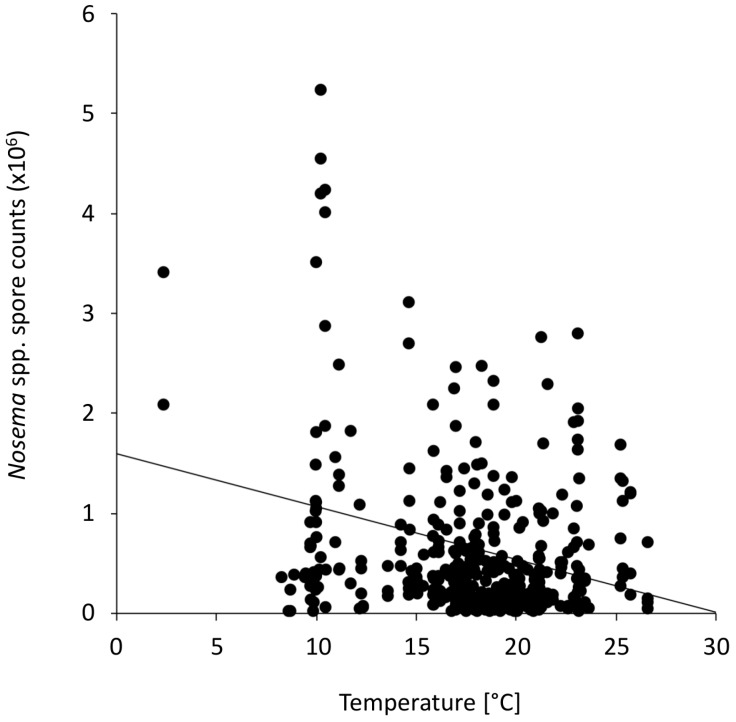
*Nosema* spp. intensities in the investigated honey bee colonies and the average monthly ambient apiary temperature. A significant negative correlation was found (*N* = 383, Spearman rank correlation, *r* = −0.1788, *p* = 0.0004). The association is displayed in a scatter plot with the data points (black points) and a trend line.

The proportion of flight days and *Nosema* spp. intensities showed significant negative correlations for all investigated time intervals prior to sampling (Spearman rank correlation, four, three and two weeks prior to sampling: all *r*s = −0.25 to −0.26, all *p*s < 0.0001, one week prior sampling: *r* = −0.13, *p* < 0.018, Table 1, Supplementary Material Dataset 2 Flight days). *Nosema* spp. intensities increased as the proportion of flight days decreased. Furthermore, *Nosema* spp. intensities in *Nosema*-positive samples from the entire study period (*N* = 383) showed a significant negative correlation with average monthly midday temperature values (*r* = −0.1788, *p* = 0.0004, Table 2, Figure 3, Supplementary Material Dataset 3 Temperature). Similar to the association above, *Nosema* spp. intensities increased as the average monthly midday temperature values decreased.

## 4. Discussion

The data revealed a significant influence of the proportion of honey bee flight days on the *Nosema* spp. intensity in workers. This outcome was consistently observed for flight day proportions measured during four, three, two and one week prior to sampling if colony was used as the experimental unit, as well as for four, three and two weeks prior to sampling on the apiary level. Additionally, flight day proportions, as well as the average ambient apiary temperature showed significant negative correlations with *Nosema* spp. intensities. The highest parasite intensities were measured in winter.

Molecular analyses confirmed previous studies [27,28], stating that *N. ceranae* is the predominant *Nosema* species in Swiss honey bee colonies. Although the two *Nosema* species exhibit differential sensitivity to temperature [16,44], the life cycles of both parasite species are similar [22,45] and also the excretion of viable spores along with the feces is most probably equal for both *Nosema* species. Therefore, we would not expect to observe different effects of ambient temperature on parasite intensity, despite the presence of *N. apis* in 10.53% of the samples.

The observed *Nosema* spp. intensities (in the range of 0.2 × 10^6^ to 4.2 × 10^6^ spores/bee) seem rather low compared to previous reports of *Nosema* spp. intensities in fully-infected individual workers (i.e., approx. 30 × 10^6^ spores/bee [46], or even up to 150 × 10^6^ spores/bee [47]). This is probably due to the quantification of the parasite intensity in pooled samples, where the presence of uninfected bees reduced the mean or median value of detected spores. Indeed, in other surveys that used pooled samples for quantification, *Nosema* spp. intensities were also considerably smaller than intensities in individual bees (e.g., [48,49]). A further aspect that makes comparison between studies more difficult are diverse applied methods [42] concerning sample collection (e.g., [50]), sample size (e.g., [51]) or spore quantification (e.g., [48,50,52,53,54]). In this context, one relevant difference between studies relates to the processing of the experimental workers; while the present study and others (e.g., [50,52,55]) used whole bees to quantify the total amount of *Nosema* spores in workers, other studies have only used midguts [33] or abdomens (e.g., [34,44]). Even though *N. ceranae* can be found using DNA methods outside of the hindgut or midgut [56], sporulation is restricted to the midguts, which have more identical limits for holding spores [57,58]. However, hindguts might contain many more spores prior to defecation. Since we measured intensity, which defines the quantity of parasites found in a given sample of hosts [13], it seemed more appropriate to use whole bees to have an accurate measure of the total numbers of parasites (=spores) in the host.

In this study, *Nosema* spp. intensities showed a trend to higher levels in winter and early spring; this seasonal pattern was consistent for both years. These findings contradict previous reports stating higher intensities in spring (e.g., [49]), but are in line with a recent study from Taiwan that found a peak of honey bee worker midgut *N. ceranae* intensity in winter, as well as a significant negative correlation between temperature and *N. ceranae* intensities under local subtropical and tropical conditions [33]. No effect of weather conditions (i.e., temperature and rainfall) on *Nosema* spp. intensities could be observed in a recent study in feral honey bees which was conducted in a U.S. region with hot summers and cool winters [34].

The tendency to higher intensities in the colder season generally supports the observed association between temperature and *Nosema* spp. intensities. The restriction of flight days by low ambient temperature correlated significantly with higher *Nosema* spp. intensities, thereby highlighting that ambient temperature may exhibit a considerable impact on parasite intensity. Observed higher *Nosema* spp. intensities in winter during our study may be explained by a reduced possibility of bees to fly and defecate during the cold period of the year; this could lead to an accumulation of spores in infected bees [36,59]. The second winter samples were taken towards the end of the winter and might reflect the bees that had at least some opportunities to fly out to defecate. Alternatively, but not mutually exclusive, highly infected individuals may have died prior to the sampling, even though mortality of *Nosema* spp. infected individuals is not elevated according to a recent study that was conducted under field-realistic conditions [60].

The potential influence of host opportunities to defecate on parasite intensity is supported by the significant differences of *Nosema* spp. intensities in bees that had 100% versus less than 100% of flight days available to them. The observation that the group of flight days with less than 100% still had a median proportion of 68% suggests that a relatively small difference in flight day proportions can influence *Nosema* spp. intensity. Indeed, if 90% and 75% were used as thresholds for flight day proportions, the differences are still significant. However, 50% of possible flight days seem to be a switching point; if the workers were able to fly out on half of the available days, the *Nosema* spp. intensities showed no difference. Calculation of flight day proportions based on the ambient temperature alone may have neglected other potentially influencing factors such as precipitation, wind, or the lack of sufficient solar radiation (e.g., [19,35]). Nevertheless, similar outcomes among the different time periods prior to sampling, similar results if apiary was used as the experimental unit, as well as the correlation between proportions of flight days and *Nosema* spp. intensities, strongly support the finding that flight days, based on the ambient temperature, exhibit a considerable influence on parasite intensity. A recent comparison of *Nosema* spp. levels in outdoor- vs. indoor-wintered hives has shown increased levels of parasites over-wintered outdoors, and a decrease in the indoor-wintered hives [61]. Similar to the above-mentioned study, previous reports of a potential impact of temperature on *Nosema* infections in bees as a result of reduced opportunities to fly out have exclusively focused on winter [62,63,64]. The observed impact of reduced flight days includes not only the extreme scenario in winter, but also the warmer months of the year.

The relationship between *Nosema* spp. intensity and the average monthly ambient apiary temperature illustrates an interesting pattern of the effects of ambient temperature. While *Nosema* spp. intensities do not greatly vary within the 15 to 25 °C temperature range, they show broader variations, and particularly high values, around or below 10 °C. This indicates that *Nosema* spp. intensities in honey bees may be mainly affected in this specific range of temperature, corresponding to the temperature threshold, where bees are considered able to fly [19].

The impact of flight days on parasite intensity and the observed association between ambient temperature and parasite intensity in honey bees suggest a distinct biological mechanism, rather than just an unspecific relationship between cold ambient temperature and *Nosema* spp. intensity. However, correlation is not causation. Therefore, the lack of defecation opportunities due to restricted flight days as the possible underlying mechanism needs to be empirically verified, e.g., via measuring flight activities of individually labeled and infected workers in combination with *Nosema* spp. spore quantification in separated midguts and hindguts.

The present study provides new knowledge of how endoparasite populations can be affected by environmental conditions, and helps to further understand parasite-host population dynamics in honey bees. The study demonstrates that low ambient temperature can be advantageous for a parasite population, assuming that higher spore loads enhance future transmission. It also highlights the importance of studying such associations in the field, and not only under laboratory conditions.

## 5. Conclusions

The present study revealed a significant effect of ambient apiary temperature on the intensity of the endoparasites *Nosema* spp. in honey bee workers. The comparison of parasite intensities among different flight day proportions (thresholds of 100%, 90% and 75%) showed significantly higher levels when bees could not fly. This observed effect of restricted flight days on parasite intensities could be the result of a lack of defecation opportunities.

## Figures and Tables

**Table 1:** Comparisons of median *Nosema* spp. intensities between different groups of flight day proportions (100%, 90%, 75%, and 50% flight day thresholds) are shown using colony as the experimental unit. The results of the Wilcoxon rank sum tests are displayed including time intervals, median *Nosema* spp. intensities, sample sizes *N* (=number of colony samples) and *p*-values. For apiary as the experimental unit, results are shown for the comparison of parasite intensities in 100% vs. <100% of flight day proportions.

Group	Time Interval	Median *Nosema* spp. Intensities (×10^6^)	*N*	*p*-Value
**Colony as the Experimental Unit**				
100% vs. <100%	4 weeks b.s. ^1^	0.28, 0.48	240, 101	0.000002 ***
	3 weeks b.s. ^1^	0.28, 0.50	241, 100	0.000002 ***
	2 weeks b.s. ^1^	0.28, 0.58	245, 96	0.000000 ***
	1 weeks b.s. ^1^	0.33, 0.44	287, 54	0.022882 *
≥90% vs. <90%	4 weeks b.s. ^1^	0.31, 0.44	279, 62	0.000564 ***
	3 weeks b.s. ^1^	0.31, 0.44	275, 66	0.000251 ***
	2 weeks b.s. ^1^	0.30, 0.48	264, 77	0.00005 ***
	1 weeks b.s. ^1^	0.33, 0.44	293, 48	0.027709 *
≥75% vs. <75%	4 weeks b.s. ^1^	0.31, 0.44	285, 56	0.000629 ***
	3 weeks b.s. ^1^	0.31, 0.45	284, 57	0.000565 ***
	2 weeks b.s. ^1^	0.31, 0.44	292, 49	0.009982 **
	1 weeks b.s. ^1^	0.34, 0.44	299, 42	0.032027 *
≥50% vs. <50%	4 weeks b.s. ^1^	0.36, 0.34	333, 8	0.489272
	3 weeks b.s. ^1^	0.36, 0.36	322, 19	0.683875
	2 weeks b.s. ^1^	0.34, 0.45	320, 21	0.09639
	1 weeks b.s. ^1^	0.34, 0.44	317, 24	0.065977
**Apiary as the Experimental Unit**				
100% vs. <100%	4 weeks b.s. ^1^	0.29, 0.49	97, 47	0.00052 ***
	3 weeks b.s. ^1^	0.31, 0.53	98, 46	0.000472 ***
	2 weeks b.s. ^1^	0.31, 0.57	100, 44	0.000157 ***
	1 weeks b.s. ^1^	0.37, 0.43	116, 28	0.205144

^1^ b.s. = before sampling; * significance level *p* < 0.05; ** significance level *p* < 0.01; *** significance level *p* < 0.001.

**Table 2:** Correlations between *Nosema* spp. intensities of the pooled honey bee worker samples and both average monthly ambient midday apiary temperature and proportions of honey bee flight days. The results of the Spearman rank correlations are shown, including time intervals and sample sizes *N* (=number of colony samples).

Correlation	Time Interval	*N*	Spearman Rank Correlation Coefficient	*p*-Value
*Nosema* spp. and temperature	whole study period	383	−0.1788	0.0004 ***
*Nosema* spp. and flight day prop. ^1^	4 weeks b.s. ^2^	341	−0.2469	3.96 × 10^−6^ ***
*Nosema* spp. and flight day prop. ^1^	3 weeks b.s. ^2^	341	−0.2485	3.4 × 10^−6^ ***
*Nosema* spp. and flight day prop. ^1^	2 weeks b.s. ^2^	341	−0.2626	8.76 × 10^−7^ ***
*Nosema* spp. and flight day prop. ^1^	1 week b.s. ^2^	341	−0.1282	0.0179 *

^1^ prop. = proportions; ^2^ b.s. = before sampling; * significance level *p* < 0.05; *** significance level *p* < 0.001.

## Supplementary Materials

Dataset 1 *Nosema* spp. intensity: *Nosema* spp. spore count data of the honey bee samples including sampling month and year; Dataset 2 Flight days: Flight day proportion data based on colonies as well as apiaries including proportions for different time periods before sampling, midday temperature measures and *Nosema* spp. spore counts; Dataset 3 Temperature: Monthly average midday temperatures for the different sampling months and corresponding *Nosema* spp. spore counts. The supplementary materials are available online at http://www.mdpi.com/2075-4450/8/1/20/s1.

## References

[1] Evans J.D., Schwarz R.S. (2011). Bees brought to their knees: Microbes affecting honey bee health. Trends Microbiol..

[2] Le Conte Y., Ellis M., Ritter W. (2010). *Varroa* mites and honey bee health: Can *Varroa* explain part of the colony losses?. Apidologie.

[3] Van engelsdorp D., Meixner M.D. (2010). A historical review of managed honey bee populations in Europe and the United States and the factors that may affect them. J. Invertebr. Pathol..

[4] Neumann P., Carreck N.L. (2010). Honey bee colony losses. J. Apic. Res..

[5] Potts S.G., Biesmeijer J.C., Kremen C., Neumann P., Schweiger O., Kunin W.E. (2010). Global pollinator declines: Trends, impacts and drivers. Trends Ecol. Evol..

[6] Wassom D.L., Dick T.A., Arnason N., Strickland D., Grundmann A.W. (1986). Host genetics: A key factor in regulating the distribution of parasites in natural host populations. J. Parasitol..

[7] Roulin A., Brinkhof M.W.G., Bize P., Richner H., Jungi T.W., Bavoux C., Boileau N., Burneleau G. (2003). Which chick is tasty to parasites? The importance of host immunology vs. parasite life history. J. Anim. Ecol..

[8] Dunn A.M., Hogg J.C., Hatcher M.J. (2006). Transmission and burden and the impact of temperature on two species of vertically transmitted microsporidia. Int. J. Parasitol..

[9] Polley L., Hoberg E., Kutz S. (2010). Climate change, parasites and shifting boundaries. Acta Vet. Scand..

[10] Fenoy S., Rueda C., Higes M., Martín-Hernández R., del Aguila C. (2009). High-level resistance of *Nosema ceranae*, a parasite of the honeybee, to temperature and desiccation. Appl. Environ. Microbiol..

[11] Le Conte Y., Navajas M. (2008). Climate change: Impact on honey bee populations and diseases. Rev. Sci. Tech. Oie..

[12] Lafferty K.D., Kuris A.M. (1999). How environmental stress affects the impacts of parasites. Limnol. Oceanogr..

[13] Rozsa L., Reiczigel J., Majoros G. (2000). Quantifying parasites in samples of hosts. J. Parasitol..

[14] Rosenkranz P., Aumeier P., Ziegelmann B. (2010). Biology and control of *Varroa destructor*. J. Invertebr. Pathol..

[15] Anderson D.L., Morgan M.J. (2007). Genetic and morphological variation of bee-parasitic Tropilaelaps mites (Acari: *Laelapidae*): New and re-defined species. Exp. Appl. Acarol..

[16] Fries I. (2010). *Nosema ceranae* in European honey bees (*Apis mellifera*). J. Invertebr. Pathol..

[17] Chen Y.P., Siede R. (2007). Honey bee viruses. Adv. Virus Res..

[18] Fries I. (1993). *Nosema apis*—A parasite in the honey bee colony. Bee World.

[19] Winston M.L. (1987). The Biology of the Honey Bee.

[20] Southwick E.E. (1983). The honey bee cluster as a homeothermic superorganism. Comp. Biochem. Phys. A.

[21] Zander E. (1909). Tierische Parasiten als Krankenheitserreger bei der Biene. Münchener Bienenztg..

[22] Fries I., Feng F., da Silva A., Slemenda S.B., Pieniazek N.J. (1996). *Nosema ceranae* n. sp. (Microspora, Nosematidae), morphological and molecular characterization of a microsporidian parasite of the Asian honey bee *Apis cerana* (Hymenoptera, Apidae). Eur. J. Protistol..

[23] Webster T.C. (1993). *Nosema apis* spore transmission among honey bees. Am. Bee J..

[24] Keeling P.J., Fast N.M. (2002). Microsporidia: Biology and evolution of highly reduced intracellular parasites. Annu. Rev. Microbiol..

[25] Malone L.A., Gatehouse H.S., Tregidga E.L. (2001). Effects of time, temperature, and honey on *Nosema apis* (Microsporidia: Nosematidae), a parasite of the honeybee, *Apis mellifera* (Hymenoptera: Apidae). J. Invertebr. Pathol..

[26] Martín-Hernández R., Meana A., García-Palencia P., Marín P., Botías C., Garrido-Bailón E., Barrios L., Higes M. (2009). Effect of temperature on the biotic potential of honeybee microsporidia. Appl. Environ. Microbiol..

[27] Dainat B., van Engelsdorp D., Neumann P. (2012). Colony collapse disorder in Europe. Environ. Microbiol. Rep..

[28] Dainat B., Evans J.D., Chen Y.P., Gauthier L., Neumann P. (2012). Predictive markers of honey bee colony collapse. PLoS ONE.

[29] Martín-Hernández R., Meana A., Prieto L., Salvador A.M., Garrido-Bailón E., Higes M. (2007). Outcome of colonization of *Apis mellifera* by *Nosema ceranae*. Appl. Environ. Microbiol..

[30] Borchert A. (1928). Beiträge zur Kenntnis des Bienenparasiten *Nosema apis*. Arch. Für Bienenkd..

[31] Bailey L. (1955). The epidemiology and control of *Nosema* disease of the honey bee. Ann. Appl. Biol..

[32] Traver B.E., Williams M.R., Fell R.D. (2012). Comparison of within hive sampling and seasonal activity of *Nosema ceranae* in honey bee colonies. J. Invertebr. Pathol..

[33] Chen Y.W., Chung W.P., Wang C.H., Softer L.F., Huang W.F. (2012). *Nosema ceranae* infection intensity highly correlates with temperature. J. Invertebr. Pathol..

[34] Rangel J., Baum K., Rubink W.L., Coulson R.N., Johnston J.S., Traver B.E. (2016). Prevalence of *Nosema* species in a feral honey bee population: A 20-year survey. Apidologie.

[35] Burrill R.M., Dietz A. (1981). The response of honey bees to variations in solar radiation and temperature. Apidologie.

[36] Hertig M. (1923). The normal and pathological histology of the ventriculus of the honey-bee, with special reference to infection with *Nosema apis*. J. Parasitol..

[37] Lotmar R. (1944). Über den Einfluss der Temperatur auf den Parasiten *Nosema apis*. Beih. Schweiz. Bienenztg..

[38] Bailey L., Ball B.V. (1991). Honey Bee Pathology.

[39] Fries I., Ekbohm G., Villumstad E. (1984). *Nosema apis*, sampling techniques and honey yield. J. Apic. Res..

[40] Pirk C.W.W., de Miranda J.R., Kramer M., Murray T.E., Nazzi F., Shutler D., van der Steen J.J.M., van Dooremalen C. (2013). Statistical guidelines for *Apis mellifera* research. J. Apicult. Res..

[41] Cantwell G.E. (1970). Standard methods for counting *Nosema* spores. Am. Bee J..

[42] Fries I., Chauzat M.P., Chen Y.P., Doublet V., Genersch E., Gisder S., Higes M., McMahon D.P., Martín-Hernández R., Natsopoulou M. (2013). Standard methods for *Nosema* research. J. Apic. Res..

[43] Van engelsdorp D., Evans J.D., Saegerman C., Mullin C., Haubruge E., Nguyen B.K., Frazier M., Frazier J., Cox-Foster D., Chen Y.P. (2009). Colony collapse disorder: A descriptive study. PLoS ONE.

[44] Gisder S., Hedtke K., Mockel N., Frielitz M.C., Linde A., Genersch E. (2010). Five-year cohort study of *Nosema* spp. in Germany: Does climate shape virulence and assertiveness of *Nosema ceranae*?. Appl. Environ. Microbiol..

[45] Higes M., García-Palencia P., Martín-Hernández R., Meana A. (2007). Experimental infection of *Apis mellifera* honeybees with *Nosema ceranae* (Microsporidia). J. Invertebr. Pathol..

[46] Paxton R.J., Klee J., Korpela S., Fries I. (2007). *Nosema ceranae* has infected *Apis mellifera* in Europe since at least 1998 and may be more virulent than *Nosema apis*. Apidologie.

[47] Aufauvre J., Biron D.G., Vidau C., Fontbonne R., Roudel M., Diogon M., Viguès B., Belzunces L.P., Delbac F., Blot N. (2012). Parasite-insecticide interactions: A case study of *Nosema ceranae* and fipronil synergy on honeybee. Sci. Rep. UK.

[48] Bourgeois A.L., Rinderer T.E., Beaman L.D., Danka R.G. (2010). Genetic detection and quantification of *Nosema apis* and *N. ceranae* in the honey bee. J. Invertebr. Pathol..

[49] Traver B.E., Fell R.D. (2011). Prevalence and infection intensity of *Nosema* in honey bee (*Apis mellifera* L.) colonies in Virginia. J. Invertebr. Pathol..

[50] Giersch T., Berg T., Galea F., Hornitzky M. (2009). *Nosema ceranae* infects honey bees (*Apis mellifera*) and contaminates honey in Australia. Apidologie.

[51] Yoshiyama M., Kimura K. (2011). Distribution of *Nosema ceranae* in the European honeybee, *Apis mellifera* in Japan. J. Invertebr. Pathol..

[52] Cox-Foster D.L., Conlan S., Holmes E.C., Palacios G., Evans J.D., Moran N.A., Quan P.L., Briese T., Hornig M., Geiser D.M. (2007). A metagenomic survey of microbes in honey bee colony collapse disorder. Science.

[53] Chen Y., Evans J.D., Zhou L., Boncristiani H., Kimura K., Xiao T., Litkowski A.M., Pettis J.S. (2009). Asymmetrical coexistence of *Nosema ceranae* and *Nosema apis* in honey bees. J. Invertebr. Pathol..

[54] Chaimanee V., Warrit N., Chantawannakul P. (2010). Infections of *Nosema ceranae* in four different honeybee species. J. Invertebr. Pathol..

[55] Medici S.K., Sarlo E.G., Porrini M.P., Braunstein M., Eguaras M.J. (2012). Genetic variation and widespread dispersal of *Nosema ceranae* in *Apis mellifera* apiaries from Argentina. Parasitol. Res..

[56] Chen Y.P., Evans J.D., Murphy C., Gutell R., Zuker M., Gundensen-Rindal D., Pettis J.S. (2009). Morphological, molecular, and phylogenetic characterization of *Nosema ceranae*, a microsporidian parasite isolated from the European honey bee, *Apis mellifera*. J. Eukaryot. Microbiol..

[57] Forsgren E., Fries I. (2010). Comparative virulence of *Nosema ceranae* and *Nosema apis* in individual European honey bees. Vet. Parasitol..

[58] Huang W.F., Solter L.F. (2013). Comparative development and tissue tropism of *Nosema apis* and *Nosema ceranae*. J. Invertebr. Pathol..

[59] Moeller F. (1978). Nosema Disease—Its Control in Honey Bee Colonies.

[60] Retschnig G., Williams G.R., Odemer R., Boltin J., Di Poto C., Mehmann M.M., Retschnig P., Winiger P., Rosenkranz P., Neumann P. (2015). Effects, but no interactions, of ubiquitous pesticide and parasite stressors on honey bee (*Apis mellifera*) lifespan and behaviour in a colony environment. Environ. Microbiol..

[61] Desai S.D., Currie R.W. (2016). Effects of wintering environment and parasite–pathogen interactions on honey bee colony loss in North Temperate regions. PLoS ONE.

[62] Moeller F.E. (1972). Effects of emerging bees and of winter flights on *Nosema* disease in honeybee colonies. J. Apic. Res..

[63] Lotmar R. (1943). Bestehen Beziehungen zwischen der Witterung und dem seuchenhaften Auftreten der Frühjahrsschwindsucht (Nosema-Amöben-Seuche)?. Schweiz. Bienenztg..

[64] Williams G.R., Shutler D., Rogers R.E.L. (2010). Effects at Nearctic north-temperate latitudes of indoor versus outdoor overwintering on the microsporidium *Nosema ceranae* and western honey bees (*Apis mellifera*). J. Invertebr. Pathol..

